# Enhanced Ethanol and Biogas Production from Pinewood by NMMO Pretreatment and Detailed Biomass Analysis

**DOI:** 10.1155/2014/469378

**Published:** 2014-08-04

**Authors:** Marzieh Shafiei, Keikhosro Karimi, Hamid Zilouei, Mohammad J. Taherzadeh

**Affiliations:** ^1^Department of Chemical Engineering, Isfahan University of Technology, Isfahan 84156-83111, Iran; ^2^Industrial Biotechnology Group, Institute of Biotechnology and Bioengineering, Isfahan University of Technology, Isfahan 84156-83111, Iran; ^3^Swedish Center for Resource Recovery, University of Borås, 501 90 Borås, Sweden

## Abstract

N-Methyl morpholine-N-oxide (NMMO) is an environmentally friendly and commercially applied cellulose solvent that is suggested for pretreatment of lignocelluloses to improve biofuel productions. However, the underlying mechanisms of the improvements have been poorly understood yet. In an attempt to investigate the mechanisms, pinewood powder and chips were pretreated with 85% (w/w) NMMO at 120°C for 1–15 h. The pretreatment improved ethanol production yield from 7.2% (g/g) for the untreated wood powder to 68.1–86.1% (g/g) and from 1.7% (g/g) for the untreated wood chips to 12.6–51.2% (g/g) of theoretical yield. Similarly, the biogas yields of untreated wood chips and powder were improved from 21 and 66 (mL/g volatile solids) by 3.5–6.8- and 2.6–3.4-folds, respectively. SEM micrographs indicated major increase in the wood porosity by the pretreatment, which would confirm increase in the water swelling capacity as well as enzyme adsorption. The analysis of X-ray diffraction showed considerable reduction in the cellulose crystallinity by the pretreatment, while FTIR spectroscopy results indicated reduction of lignin on the wood surface by the pretreatment.

## 1. Introduction

Lignocelluloses are promising raw materials for production of second generation of ethanol. These relatively cheap and abundant materials may solve the food versus fuel conflict which is a result of production of biofuels from sugar and starch based materials [1, 2]. The process for production of the first generation of bioethanol involves hydrolysis, fermentation, distillation, and dehydration. However, a major challenge of using lignocelluloses is their recalcitrance to saccharification and poor hydrolysis yields. Thus, prior to enzymatic hydrolysis, a pretreatment step is necessary to open up this structure and improve the product yields as well as the process of economy [1, 3, 4]. Similarly, the bottleneck of biogas production process from lignocelluloses is the first step of anaerobic digestion, that is, the rate-limiting hydrolysis step. An efficient pretreatment step can eliminate this bottleneck [1, 3, 4]. In native cell wall of woody biomass, cellulose chains are packed into micro- and macrofibers. Hemicelluloses and lignins are present in the matrix surrounded celluloses and form covalent and noncovalent bindings to cellulose and each other. The whole reinforced composite of the cell wall acts as the main barriers to the production of monomer sugars. Another barrier is the high concentration (50–90%) of lignin in the spaces between cells, that is, cell lamella. Lignin is highly hydrophobic and also inhibits activity of cellulases. Pretreatment can effectively overcome these obstacles by rearranging cellulose, hemicellulose, and lignin to a less recalcitrant structure. Pretreatment can also increase the micro- and macroaccessibility of the hydrolyzing enzymes to the biomass [5]. Among all pretreatment methods, treatment with cellulose solvents such as ionic liquids (ILs) [4, 6, 7], phosphoric acid [8], NaOH/urea [9], and N-methylmorpholine-N-oxide (NMMO) [6, 10, 11] is well known for their high efficiencies. N-Methylmorpholine N-oxide (NMMO) is an industrial cellulose solvent for fiber making, and pretreatment with this green chemical produces no toxic wastes. This solvent can be almost completely recycled and reused several times [11]. NMMO monohydrate dissolves cellulose via formation of strong hydrogen bonds. These bonds are then broken after addition of the antisolvent, for example, water. As a consequence of the pretreatment, inter- and intrachain hydrogen bonds of cellulose are altered. Thus, the regenerated cellulose is more susceptible to saccharification.

It was previously shown that NMMO pretreatment improved the yields of ethanol from spruce and oak up to 89 and 85.4%, respectively [11]. Furthermore, ethanol production from rice straw [6] and sugarcane bagasse [17] with NMMO pretreatment were studied. This pretreatment was also considered for biogas production from rice straw, triticale straw, and spruce wood [18] as well as birch wood [10]. However, to our knowledge, no previous work on ethanol and biogas production of the NMMO treated pinewood powder and chips has been presented in the literature.

In this study, NMMO pretreatment for ethanol and biogas production enhancement from pinewood powder and chips were investigated. Further experiments were conducted to understand the underlying mechanism of the improvements. Effects of the pretreatment on the wood properties were studied by SEM imaging, cellulose enzyme accessibility, and water swelling capacity. Moreover, the changes in the crystallinity of the substrate were tracked using X-ray diffraction as well as FTIR spectroscopy.

## 2. Materials and Methods

### 2.1. Raw Materials and Their Analysis

Pinewood,* Pinus eldarica*, from the forest of Isfahan University of Technology campus (Isfahan, Iran), was used for the biofuel production. After debarking and cutting, a fraction of the wood was milled and screened to achieve a wood powder with particles of less than 1 mm. A fraction was also used as wood chips with approximate dimensions of 0.5 by 0.5 by 0.1 centimeters. The wood was analyzed for carbohydrate and lignin contents according to the standard procedure described by Sluiter et al. [19].

### 2.2. Pretreatment

A commercial grade of NMMO solution (50%, BASF, Germany) was concentrated to 85% (w/w) solution by vacuum evaporation. An amount of 15 grams of the wood was pretreated by 185 grams of the NMMO solution according to the method described by Shafiei et al. [11]. Briefly, NMMO solution was added to preheated beakers containing wood and mixed by glass rods. The beakers were kept in an oil bath for 1, 3, and 15 hours at 120°C. Afterwards, 150 mL boiling distilled water was added to each of the pretreatment suspensions. The materials were then washed and recovered by vacuum filtration. Freeze-drying was used for dry weight measurement of the pretreated woods at −48°C for 48 h. Similar to the untreated wood, the carbohydrate and lignin contents of the pretreated materials were measured. The pretreated materials were kept in sealable bags at 4°C until use.

### 2.3. Enzymatic Hydrolysis

The treated and untreated woods were subjected to enzymatic hydrolysis using 15 FPU cellulase (Celluclast 1.5 L, Novozyme, Denmark) and 30 IU *β*-glucosidase (Novozyme 188, Novozyme, Denmark) per gram of substrate. The hydrolysis was performed at 45°C for 96 h using 5% (w/w) wood (based on the dry weight) in 50 mM sodium citrate buffer (pH 4.8) and 0.4 g/L sodium azide [4]. Sodium azide was added as a metabolite inhibitor in the hydrolysis. Since sodium azide inhibits fermentation, a parallel set of enzymatic hydrolysis without addition of sodium azide was conducted to be used in the fermentation. This hydrolysis was carried out without sample taking to minimize contaminations. The activities of cellulase and *β*-glucosidase were measured to 70 FPU/mL and 220 IU/mL, respectively, based on the methods described by Adney and Baker [20] and Ximenes et al. [21]. The yield of enzymatic hydrolysis was calculated as (g) produced glucose by hydrolysis/(g) glucan in the biomass/1.111∗100. The recovery of solid after pretreatment was not considered in the calculations. The glucan content for each wood sample (native or pretreated at different condition) prior to enzymatic hydrolysis was considered for calculation of the yield of the sample.

### 2.4. Ethanol Fermentation

The liquid fraction, the hydrolysate, was separated by centrifugation under aseptic conditions and supplemented with essential nutrients for the cells. Then, fermentations were performed in 50 mL centrifuge tubes at 32°C for 24 h using high cell density of a flocculating strain of* Saccharomyces cerevisiae* (CCUG 53310, Culture Collection of University of Gothenburg, Sweden). The method used for strain maintenance and inoculum preparation was described by Shafiei et al. [4, 11]. The yield is calculated based on the grams of ethanol produced per grams of ethanol which can theoretically be produced from glucan in each of the wood samples prior to enzymatic hydrolysis. Thus, ethanol yield equals to (g) ethanol in fermentation broth/(g) glucan in the sample/1.111/0.51∗100.

### 2.5. Biogas Production

Anaerobic digestion was performed in batch digesters using mesophilic bacteria from a 7000 m^3^ biogas digester of Isfahan Municipal Wastewater Treatment (Isfahan, Iran). The inoculum contains bacterial consortia for biogas production which operates at 37°C. Serum glass bottles were filled with 20 mL of inoculum, 0.25 g of the treated or untreated wood, and 5 mL of deionized water and closed with butyl rubber seals and aluminum caps [9]. The digestion was conducted at 37°C for 45 days. Gas mixture containing 80% nitrogen and 20% carbon dioxide was used for initial flushing the bottle's headspace to obtain anaerobic conditions. Water and inoculum were used as a control in order to determine the biogas production of the inoculum. Gas samples were taken from the headspace of the bottles and analyzed for methane and carbon dioxide content by gas chromatography.

### 2.6. Structural Analysis

#### 2.6.1. Scanning Electron Microscopy (SEM)

The microscopic structure of the treated and untreated wood powder was determined by SEM. The dried samples were coated with gold (BAL-TEC SCD 005) and analyzed using SEM microscope (Zeiss, Germany) at 7.5 kV.

#### 2.6.2. Water Swelling Capacity and Enzyme Adsorption

Water swelling capacity, the amount of water adsorbed by the wood, was measured for the untreated and pretreated woods. An amount of 0.1 gram of the samples was put in small bags of nonwoven materials and immersed in water for one hour. Then, the swelling capacity was measured as (*w*_2_ − *w*_1_)/*w*_1_, in which *w*_1_ is the weight of the dry materials and *w*_2_ is the final weight of the swollen materials [22].

The cellulase adsorption was evaluated in 15 mL centrifuge tubes containing 1% (w/w) wood sample and 400 mg cellulase per gram of glucan, based on the method developed by Kumar and Wyman [23]. The tubes were shaken at 60 rpm for 2 hours. Then, the tubes were centrifuged for 15 minutes at 4000 rpm. The supernatants were analyzed for protein content based on the Biuret method [24].

#### 2.6.3. X-Ray Diffraction

The crystallinity of cellulose in the treated and untreated wood powder was analyzed using X-ray powder diffraction pattern of the samples. The diffractometer (Philips, X'pert, Netherlands) operated at 40 kV and 30 mA, and the spectra were collected in the range of 2*θ* = 10–30° with step size of 0.05° and step of 1 s. The crystallinity was calculated according to the method of Segal et al. [25], using CrI = ((*I*_002_ − *I*_am_)/*I*_am_)∗100, in which *I*_002_ is the intensity of the peak corresponding to crystalline portion of biomass (cellulose) at position of 2*θ* = 22.5° and *I*_am_ corresponds to amorphous portion (i.e., hemicellulose, lignin, and cellulose) at position of 2*θ* = 18° (Figure 5) [26].

#### 2.6.4. FTIR Spectroscopy

The chemical bonds and the crystallinity of the treated and untreated pinewood powder were investigated using a FTIR spectrometer (Bruker) equipped with a universal ATR (Attenuated Total Reflection) accessory and Deuterated triglycine sulfate (DTGS) detector (Bruker Tensor 27 FT-IR). The spectra were collected over using average of 60 scans and resolution of 4 cm^−1^ and at the range of 600–4000 cm^−1^. Rubberband correction method was used for correction of the spectra baseline [27], and the absorbance values were normalized to 0 and 1 based on the intensity of the maximum peak. Total crystallinity index (TCI = *a*_1377_/*a*_2922_) and lateral order index (LOI = *a*_1421_/*a*_893_) as well as the lignin to cellulose ratio (*a*_1510_/*a*_900_) were determined using the absorption ratios [12, 28].

### 2.7. Analytical Methods

The composition of metabolites of the hydrolysis and fermentation experiments were measured by HPLC equipped with a RI detector (Jasco International Co., Tokyo, Japan). Sugars were analyzed using an ion-exchange Aminex HPX-87P column (Bio-Rad, Richmond, CA, USA), while ethanol, glycerol, and other metabolites were analyzed using Aminex HPX-87H column (Bio-Rad, Richmond, CA, USA) according to the method described by Shafiei et al. [11]. The methane and carbon dioxide contents of the biogas were measured using a gas chromatograph (Sp-3420A, Propack Q column, TCD detector, Beijing, Beifen Ruili Analytical instrument Co). Helium at flow rate of 25 mL/min was used as a carrier of gas. The column, injector, and detector temperature were controlled at 50, 90, and 140°C, respectively.

Except for the triplicate biogas production experiments, all other experiments were performed in duplicates, and the results are presented as averages of the obtained data.

## 3. Results and Discussion

Pinewood powder and chips were treated with NMMO at 120°C. The treated and untreated materials were then enzymatically hydrolyzed for 96 h. Afterwards, the hydrolysates were fermented with a flocculating strain* Saccharomyces cerevisiae* for 24 h. Furthermore, the materials were anaerobically digested for biogas production.

### 3.1. Effect of Pretreatment on the Wood Composition

The compositions of untreated and pretreated wood are presented in Table 1. Glucan content was slightly increased (2–5%) after the pretreatments (Table 1). It can be explained by the loss of the hemicellulosic carbohydrates, that is, xylan (0.8–1.2%) and mannan (1.8–3.2%), by the pretreatment. The hemicelluloses decreased to a higher extent as the pretreatment time increased (Table 1). Overall, the results indicated minor changes in the composition of the treated materials, which is in line with previous studies [4, 6, 11].

### 3.2. Enzymatic Hydrolysis and Ethanol Production

**Figure 1:**
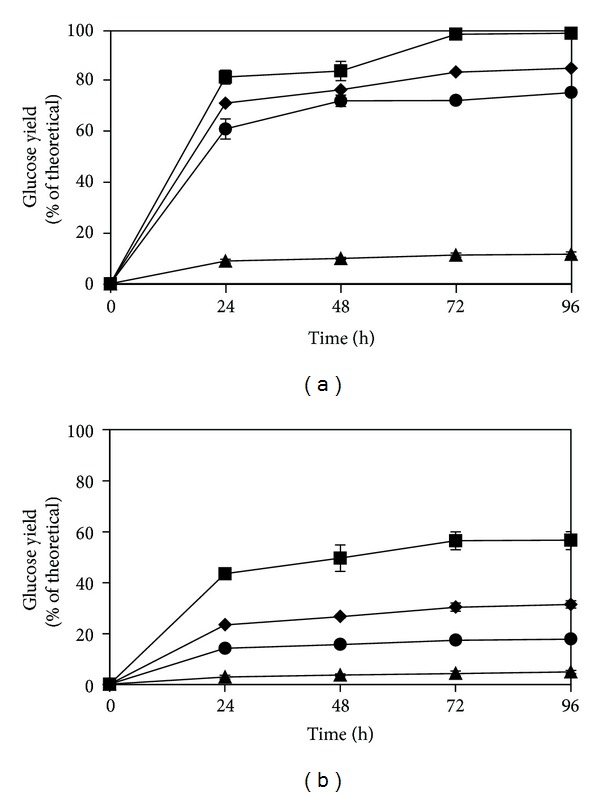
Effects of different pretreatment time and wood size on the hydrolysis yield (the yield of enzymatic hydrolysis was calculated as (g) glucose after hydrolysis/(g) glucan in the untreated or pretreated sample/1.111∗100) of pinewood powder (a) and chips (b). The symbols correspond to 1 h (●), 3 h (◆), and 15 h (■) pretreatment, and (▲) corresponds to for the untreated wood.

The effects of pretreatment on the enzymatic hydrolysis yield of pinewood powder and chips are depicted in Figure 1. Significant improvement in the glucose production yields were achieved by the pretreatment (Figure 1). The theoretical glucose yield of untreated wood powder was improved from 11.5% to 74.8–98.9%. The untreated wood chips had a theoretical glucose yield of 4.8% that was improved to 17.7–56.5% by the pretreatment at 120°C for 1–15 h. More enhancements in the saccharification yields were observed with longer pretreatment times, and the maximum yield was achieved by 15 h treatment. However, prolongation of the hydrolysis from 72 to 96 h did not affect the hydrolysis yields.

The high yields of enzymatic hydrolysis are obtained regardless of minor changes in the wood composition after the pretreatment. These data suggest that the improvements in the hydrolysis yields were not due to lignin or hemicellulose removal, but other mechanisms were responsible for the enhancements. The pretreatment was more effective on the wood powder than the wood chips. Pretreatment of the chips for 15 h improved the hydrolysis yield by 11.8-folds, while the improvement for the powder was only 8.6-folds.

**Figure 2:**
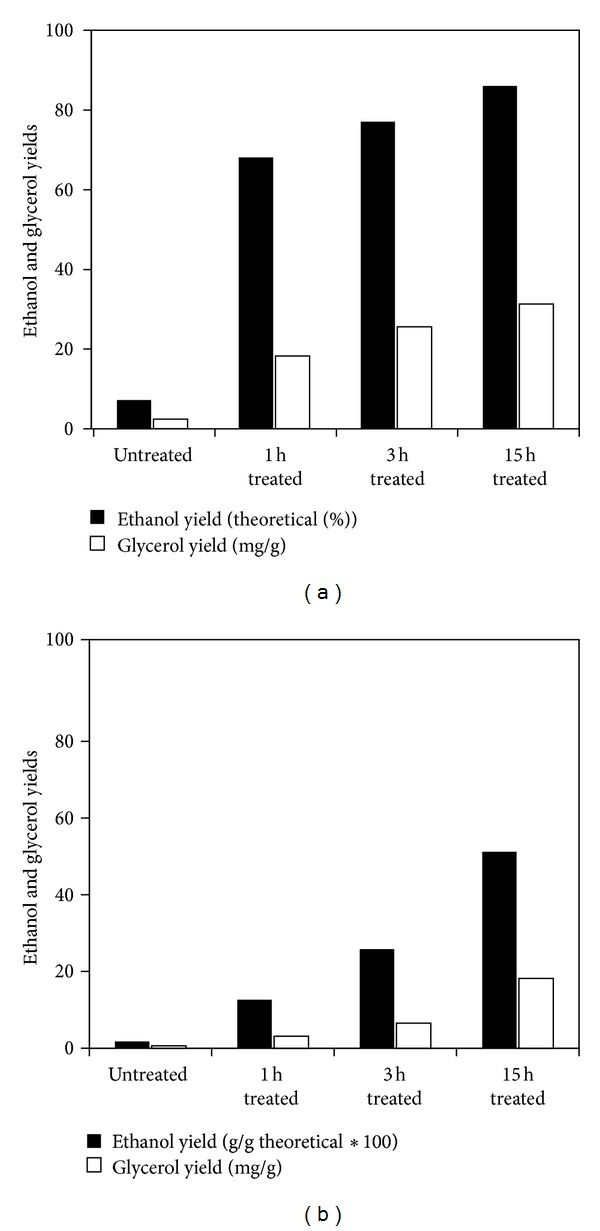
Effects of different pretreatment time and wood size on the ethanol (ethanol yield equals (g) ethanol produced/(g) glucan in the un- or pretreated sample/1.111/0.51∗100) and glycerol (milligrams of produced glycerol per gram of glucose that can theoretically be produced from the glucan in the treated or nontreated woods) yield of pinewood powder (a) and chips (b).

Significant improvement in the ethanol yield was observed by the pretreatments (Figure 2). While the theoretical ethanol yields from untreated wood chips and powder were 1.7% and 7.2%, respectively, the pretreatment improved the yields to respective values of 12.6–51.2% and 68.1–86.1%. More improvements in the ethanol yields were achieved by increasing the pretreatment duration. These improvements were more significant for the wood chips. The ethanol yield of 1 h treated chips and powder was 12.6% and 68.1%, respectively; however, 15 h treatment improved these values by 4.1- and 1.3-folds. Similar improvements in the ethanol and saccharification yield of wood chips and powder were observed in the previous studies [4, 11]. The best yields are comparable with the maximum theoretical ethanol yield of 84–90% obtained by fermentation of pure glucose by similar strain of* S. cerevisiae* [11].

### 3.3. Biogas Production from Pretreated Pinewood

**Figure 3:**
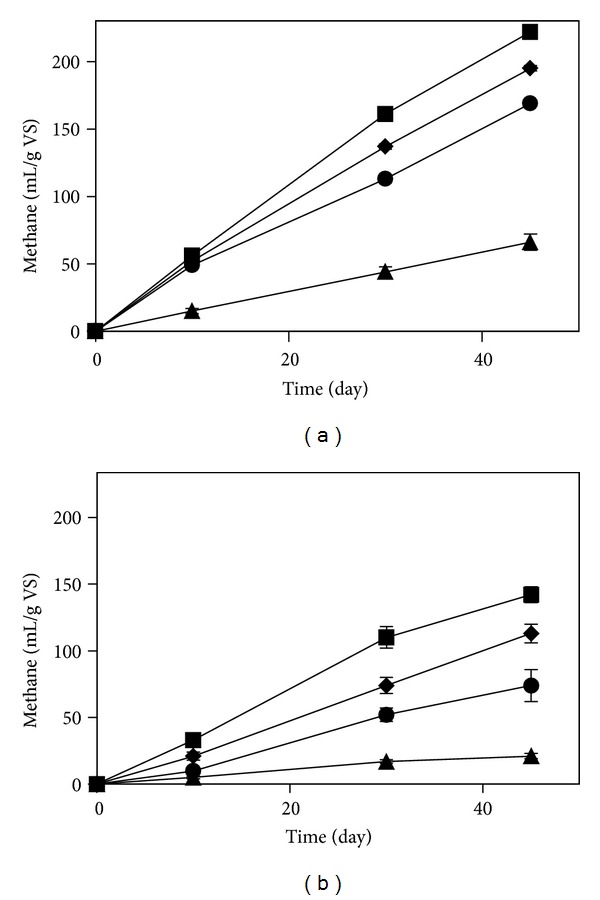
Effects of different pretreatment time and wood size on the methane yield of pinewood powder (a) and chips (b). The symbols correspond to 1 h (●), 3 h (◆), and 15 h (■) treatment, and (▲) corresponds to untreated wood. VS stands for volatile solids.

The pretreatment considerably improved the methane yield of the treated wood powder and chips (Figure 3). The amount of methane produced from the untreated wood chips and powder were 21 and 66 mL/g volatile solid (VS). However, the methane produced from 1, 3, and 15 h treated wood chips increased by 3.5-, 5.4-, and 6.8-folds, respectively. Moreover, the methane yield of 1, 3, and 15 h treated wood powder increased by 2.6-, 3-, and 3.4-folds, respectively. Increase in the pretreatment duration positively enhanced the methane yields for both wood chips and powder.

### 3.4. Structural Analysis

#### 3.4.1. Scanning Electron Microscopy (SEM)

**Figure 4:**
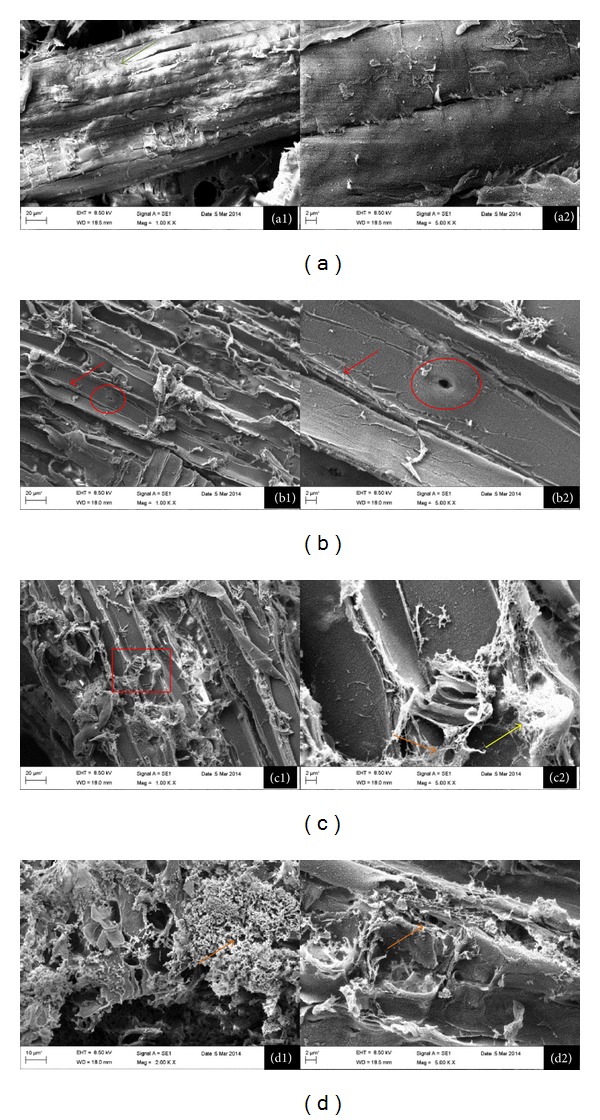
Scanning electron micrographs of the untreated (a) and NMMO treated pinewood powder for 1 h (b), 3 h (c), and 15 h (d). The magnifications were ×5000 (series 2) and ×1000 (series 1) except for (d1) which is ×2000. The arrows show the fiber bundle (green), opened cell wall (red), pores to the inside of biomass (orange), and condensation of cellulose (yellow). The cell wall pits are shown by red circles.

The structural modifications of pinewood powder after pretreatment with NMMO were investigated using SEM, and some of the captured images are presented in Figure 4. The untreated pine powder fibers had a highly compact structure, which was altered to a more accessible structure after the pretreatment. As the pretreatment time increases, more changes in the accessibility to the fibers were observed. The porous materials on the fiber surface of the pretreated wood were previously observed in the treated cellulosic materials. The condensed materials were cellulose microfibrils in the wood chemical or mechanical pulp [29, 30]. For the AFEX treated corn stem, lignin aromatics and hemicellulose oligomers with high and low molecular weight as well as decomposition products of AFEX pretreatment were observed on the outer cell walls [31]. In current study, the ability of NMMO as a cellulose dissolution agent suggests that the porous materials on the fiber surface might be the dissolved cellulose formed by condensation (Figures 4(c2), 4(d1), and 4(d2)). The increase in the ratio of cellulose/lignin on the surface of pretreated wood (Section 3.4.4) confirms the condensation of cellulose on the wood surface after the pretreatment. The cellulose which is condensed after the regeneration has an amorphous form and there is no protecting layer of cell wall matrix around it. Thus, the hydrolysis of the regenerated cellulose is much more convenient compared to the crystalline cellulose inside the cell wall.

While the fiber bundle of wood is intact in the untreated wood (Figures 4(a1) and 4(a2)), the pretreatment obviously opened up the bundle and the cell wall. Therefore, the cell walls and pits (holes on the cell wall for exchange of materials) are visible in Figures 4(b1) and 4(b2). Furthermore, the pores with 2-micrometer diameter are clearly seen in the 3 h and 15 h treated wood powder, while they are missing on the surface of the untreated wood (Figures 4(d1) and 4(d2) versus 4(a1) and 4(a2)). Thus, increase in the porosity as well as increased access to inside of fibers and cells after the pretreatment could be one of the main reasons for the enhanced yields of enzymatic hydrolysis.

Increase in the cellulose accessible surface plays an important role in the improvement of the anaerobic digestion yields. In anaerobic digestion, the size of cellulose-degrading bacteria is in the order of micrometers, and most of these bacteria should be attached to the cellulose surface and produce cellulosome enzymes which are placed on the cell surface [1]. Thus, the increased number of pores and the damaged structure of cell walls help the hydrolyzing bacteria to act more efficiently on the pretreated wood.

#### 3.4.2. Water Swelling Capacity and Enzyme Adsorption

Water swelling capacity of the treated wood powder and chips was measured, and the results are shown in Table 2. Water swelling capacity of the untreated wood chips and wood powder were 1 and 7.2 (g water/g cellulose), respectively. The pretreatment of the wood chips and powder for 1–15 h increased the swelling capacity to 95–273% and 38–53%, respectively. Moreover, increase in the pretreatment duration resulted in increase of the swelling capacity.

The adsorption of cellulase enzyme on the wood was also measured and presented as relative values (g protein adsorbed/g protein adsorbed by untreated pine chips) (Table 2). Similar to water swelling capacity, increasing trends were observed after the pretreatment with NMMO. This indicates that water swelling capacity and enzyme adsorption are directly related; the sample with higher water adsorption can bind to more enzyme molecules. However, the enzyme adsorption of the wood chips was less than that of wood powder. Furthermore, increasing the pretreatment time increased the adsorption of enzyme on both pine chips and powder. These results are in accordance with the yields of enzymatic hydrolysis of the treated materials, suggesting that more cellulase adsorption results in the higher hydrolysis and fermentation yields [22]. Considering the SEM micrographs (Figure 4), the increases in the water swelling capacity and enzyme adsorption capacity of the pretreated wood can be related to the increase in the wood porosity and enzyme accessibility.

#### 3.4.3. X-Ray Diffraction

**Figure 5:**
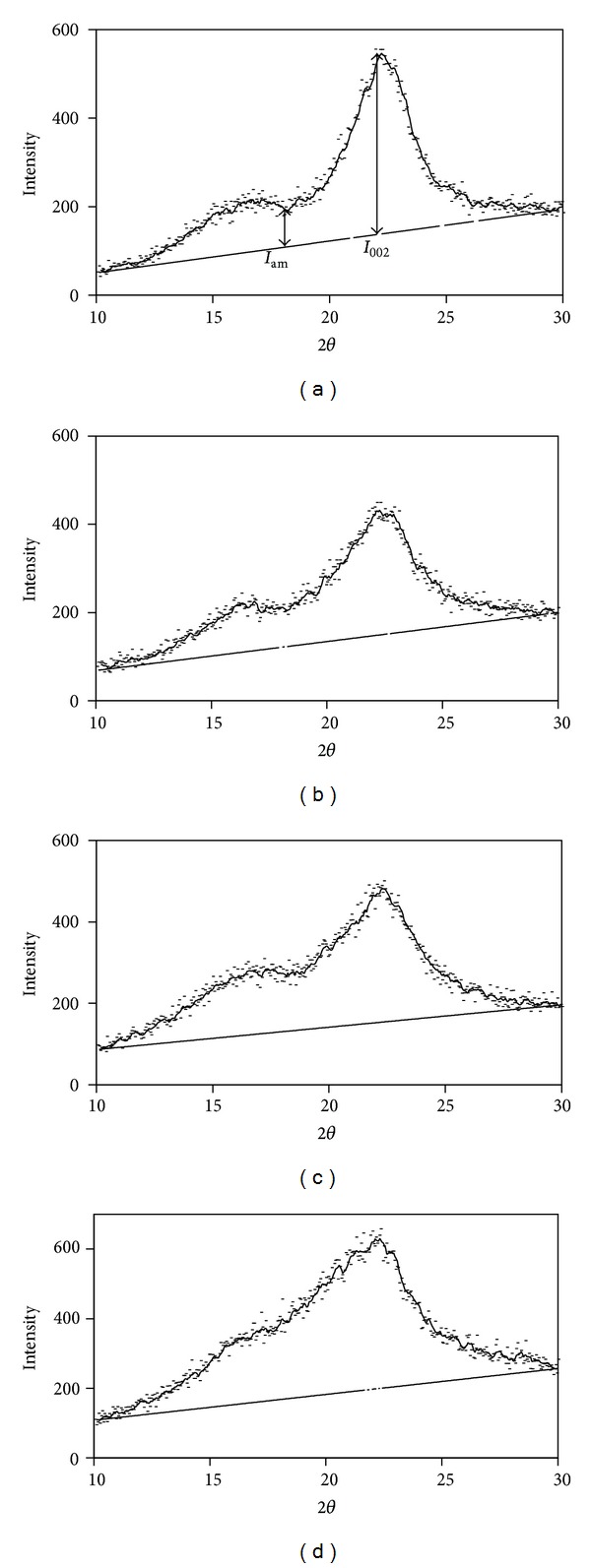
X-ray powder diffraction spectra of untreated (a) and NMMO treated pinewood powder for 1 h (b), 3 h (c), and 15 h (d). The trendline of six-point average is presented as the solid line in the spectra, and the base line is drawn from end to end of the spectra.

The changes in the crystallinity of the untreated and NMMO treated wood are presented in Table 3. Measurement of crystallinity by X-ray diffraction indicates reduction in the crystallinity of wood after pretreatment. The crystallinity reduces to a higher extent as the pretreatment time increases (Figure 5). Table 3 shows the comparison of these results with the values from FTIR spectroscopy. TCI (FTIR) represents the crystallinity of celluloses I and II, while LOI (FTIR) refers exclusively to cellulose I [28]. The crystallinity values obtained from X-ray confirm the reduction of crystallinity after the pretreatment; however, the changes in the TCI and LOI are not significant. This comparison suggests that the calculation of crystallinity of the wood by FTIR spectroscopy might not result in values consistent with the X-ray spectroscopy. The crystallinity measured by FTIR refers to the crystallinity of cellulose. Thus, the presence of hemicellulose and lignin interferes in the measurements. On the other hand, crystallinity, obtained from X-ray diffraction, is the crystallinity of the whole biomass and not cellulose alone [32]. Therefore, it can be concluded that the crystallinity of the whole biomass is reduced by pretreatment.

#### 3.4.4. FTIR Spectroscopy

**Figure 6:**
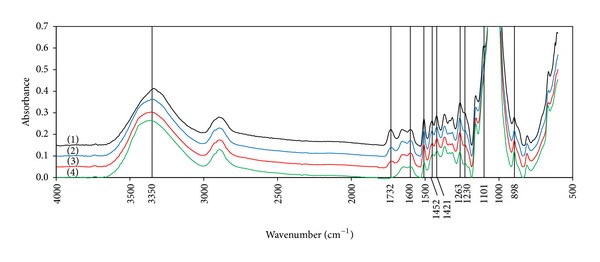
The FTIR spectra of untreated (1) and NMMO treated pinewood powder for 1 h (2), 3 h (3), and 15 h (4).

The FTIR spectra of the untreated and NMMO treated wood powder are shown in Figure 6. FTIR spectroscopy is widely used for structural analysis of cellulose and lignocellulosic materials. The absorption bands and their assigned chemical functional groups are presented in Table 4. Based on the literature data, the absorption bands at 1600, 1508, and 1263 are assigned to the functional groups of lignin [6]. The reduction in the peak intensities at 1508 cm^−1^ (from 0.12 to 0.07) and 1263 cm^−1^ (from 0.2 to 0.12) (Table 4) as a consequence of pretreatment indicates reduction of the lignin, especially the guaiacyl type, on the surface of the treated samples. It also confirms that exposure of the wood to NMMO for a longer time results in more reduction of lignin from the wood surface.

The bands at 1732 and 1230 shows the reduction of lignin and hemicellulose on the wood surface by the pretreatment, while no specific and individual band for hemicellulose reduction is observed.

Cellulose type I is the typical crystalline form of cellulose in the native plant cell wall, while the regenerated celluloses are in the form of cellulose type II and amorphous cellulose [12]. The intensity of the spectra for crystalline cellulose type I at 3352, 1452, 1431, 1162, 1111, and 893 cm^−1^ did not significantly change. The peak for the untreated wood at 3350 cm^−1^ is shifted to 3370 cm^−1^ by the pretreatment. The peak for cellulose II at 1470 is missing in all samples probably due to the presence of lignin and hemicellulose. Unexpectedly, the peak at 1420 cm^−1^ that is assigned to cellulose II was decreased. At this wave number, the functional groups assigned to lignin and hemicellulose seemed to be more effective than those of cellulose II; hence, the reduction in the peak intensity could be because of reduction in the lignin and hemicellulose content of the wood, rather than cellulose II. Another band assigned to cellulose I is at 1111 cm^−1^ which was clearly disappeared in the NMMO treated samples.

Considering all changes in the FTIR spectra of the wood after NMMO pretreatment, it could be concluded that NMMO altered the distribution of biomass matrix components, for example, condensation of amorphous cellulose on the NMMO treated wood surface and thus reduction in the surface lignin. Furthermore, the regeneration of cellulose I to cellulose II and amorphous cellulose was also possible. However, the wood has a very complex structure, and study of the effects of NMMO pretreatment on pure lignin, cellulose, and hemicellulose by FTIR may help the better understanding of the structural changes by the treatment.

## 4. Conclusions

Pinewood had a highly recalcitrant structure and the conversion of native pinewood to ethanol and biogas was inefficient. NMMO pretreatment could efficiently improve the yields of ethanol up to 86% of the theoretical yield and the biogas yield up to 222 (mL/g VS) from pinewood. No cellulose loss and minimal compositional changes were among the features of the pretreatment with NMMO. One of the main findings of the current work was that physical removal of lignin and hemicellulose is not necessary to obtain a high cellulose hydrolysis yield. Structural analyses suggested that the improvements were related to increase in the microstructure porosity, reduction in the surface lignin, and decrease in the crystallinity of cellulose. The cellulose solvent NMMO could be a potential reagent for effective pretreatment of lignocellulosic materials in the commercial scale, although the process economy should be considered.

## Figures and Tables

**Table 1:** Lignin and carbohydrate contents of untreated and NMMO treated pinewood^1^.

Pretreatment time	Glucan (%)	Mannan (%)	Xylan (%)	Galactan (%)	Arabinan (%)	ASL^2^ (%)	AINSL^3^ (%)
Untreated	41.6 ± 0.5	11.9 ± 0.5	6.1 ± 0.2	1.7 ± 0.2	0.72 ± 0.04	0.6 ± 0.02	26.0 ± 0.4
1 h treated wood	43.1 ± 0.6	10.1 ± 0.3	5.3 ± 0.1	1.7 ± 0.3	0.72 ± 0.04	0.8 ± 0.04	27.0 ± 0.5
3 h treated wood	44.0 ± 0.5	9.6 ± 0.6	5.4 ± 0.2	1.6 ± 0.2	0.74 ± 0.05	0.9 ± 0.02	27.1 ± 0.3
15 h treated wood	46.4 ± 0.8	8.7 ± 0.4	4.9 ± 0.1	1.5 ± 0.2	0.61 ± 0.07	1.5 ± 0.1	27.8 ± 0.6

^1^All data are presented on dry weight basis (treated or untreated). All the given values are means of three determinations ± standard error.

^2^Acid soluble lignin.

^3^Acid insoluble lignin.

**Table 2:** Water swelling capacity and relative enzyme adsorption of the NMMO pretreated and untreated pinewood.

Pretreatment	Wood size	Water swelling capacity (g water/g wood)^1^	Relative enzyme adsorption^2^
Untreated	Powder	7.2	4.0
Pretreated for 1 h	Powder	10.0	4.9
Pretreated for 3 h	Powder	10.7	5.2
Pretreated for 15 h	Powder	11.0	6.7

Untreated	chips	1.0	1.0
Pretreated for 1 h	chips	2.0	1.5
Pretreated for 3 h	chips	3.7	2.7
Pretreated for 15 h	chips	3.8	4.3

^1^The standard deviation of all samples was less than 6.3%.

^2^The standard deviation of all samples was less than 7.2%.

**Table 3:** The crystallinity of untreated and NMMO treated pinewood powder using FTIR and X-ray spectroscopy.

	Untreated	1 h treated	3 h treated	15 h treated
FTIR				
LOI (*a*_1421_/*a*_898_)	1.06	1.13	1.05	1.00
TCI (*a*_1375_/*a*_2918_)	1.10	1.08	1.16	1.21
Lignin/cellulose (*a*_1508_/*a*_898_)	0.94	0.99	0.83	0.55
X-ray diffraction	0.80	0.71	0.59	0.49

**Table 4:** Characteristic frequencies and band intensities from the FTIR spectra of the treated and untreated pinewood powder.

Frequency (cm^−1^)	Functional group/band assignment	Untreated powder	1 h treated	3 h treated	15 h treated	Reference
3352 (3447) (no band)	–OH stretching intramolecular hydrogen bonds Cellulose I (3352), cellulose II (3447) Xylan	0.26 (0.18)	0.26 (0.22)	0.25 (0.22)	0.26 (0.24)	[12] [12] [13]

1732	C–O stretching of acetyl or carboxylic acid Hemicellulose and lignin (1730)	0.07	0.04	0.03	0.00	[6]

1600	C=C Lignin	0.07	0.07	0.06	0.05	[6, 14]

1508	C–C stretching of the aromatic ring, lignin (1510)	0.12	0.12	0.10	0.07	[6]

1452	–OH in plane bending Cellulose I (1455), cellulose II (1470) Asymmetric bending in C–H_3_ (1465) lignin –C–H deformation Xylan (1461)	0.11	0.11	0.11	0.11	[12] [6] [13]

1421	C–H_2_ symmetric bending Cellulose II (1419), cellulose I (1431) Weak C–O stretching (1420), aromatic C=C stretch (1433) Lignin (1423), Xylan (1420) [13]	0.14	0.14	0.13	0.13	[15] [13] [13] [13]

1263	Vibration of guaiacyl rings (1270)	0.20	0.18	0.16	0.12	[14]

1230	C–O stretching in lignin and hemicel. (1235)	0.15	0.12	0.10	0.07	[16]

1101	Ring asymmetric stretching Cellulose I (1111) Cellulose II (1007)	0.46	0.44	0.44	0.49	[12]

898	Asym., out of phase ring stretching Cellulose I (893) Cellulose II, Amorphous Cellulose (895) Xylan (899)	0.13	0.12	0.12	0.12	[15] [15] [13]
